# Invasive Bacterial Diseases in Northern Canada

**DOI:** 10.3201/eid1401.061522

**Published:** 2008-01

**Authors:** Naushaba Degani, Christine Navarro, Shelley L. Deeks, Marguerite Lovgren, the Canadian International Circumpolar Surveillance Working Group

**Affiliations:** * Hospital for Sick Children, Toronto, Ontario, Canada; † Public Health Agency of Canada, Ottawa, Ontario, Canada; ‡ National Centre for Streptococcus, Edmonton, Alberta, Canada; 1 Current affiliation: National Centre for Immunisation, Research and Surveillance, Westmead, New South Wales, Australia

**Keywords:** Pneumococcal infections, Arctic regions, pneumococcal vaccines, Streptococcus pneumoniae, Haemophilus influenzae, streptococcal infections, Canada, surveillance, research

## Abstract

International Circumpolar Surveillance (ICS) is a population-based invasive bacterial disease surveillance network. Participating Canadian regions include Yukon, Northwest Territories, Nunavut, and northern regions of Québec and Labrador (total population 132,956, 59% aboriginal). Clinical and demographic information were collected by using standardized surveillance forms. Bacterial isolates were forwarded to reference laboratories for confirmation and serotyping. After pneumococcal conjugate vaccine introduction, crude annual incidence rates of invasive *Streptococcus pneumoniae* decreased from 34.0/100,000 population (1999–2002) to 23.6/100,000 population (2003–2005); substantial reductions were shown among aboriginals. However, incidence rates of *S. pneumoniae*, *Haemophilus influenzae*, and group A streptococci were higher in aboriginal populations than in non-aboriginal populations. *H. influenzae* type b was rare; 52% of all *H. influenzae* cases were caused by type a. Data collected by ICS contribute to the understanding of the epidemiology of invasive bacterial diseases among northern populations, which assists in formulation of prevention and control strategies, including immunization recommendations.

## Abstract 2

Data collected by International Circumpolar Surveillance contribute to understanding the epidemiology of these diseases.

The circumpolar region of Canada is a sparsely populated area of 1.74 million square miles comprising 3 territories (Yukon, Northwest Territories, and Nunavut) and the northern regions of Québec and Labrador. The estimated population is 132,956, which represents 0.4% of the Canadian population (*1*). The circumpolar population is younger (Table 1) and has a larger proportion of aboriginal persons than the general Canadian population. Approximately 59% of the population in the region self-identify as Inuit, First Nations, or Métis, compared with 3.3% of the total Canadian population (*2*). Northern populations tend to have higher rates of invasive bacterial diseases, including those caused by *Streptococcus pneumoniae* and *Haemophilus influenzae*, with aboriginal persons at greatest risk for disease (*3*–*5*).

**Table 1:** Total and age-specific population estimates for the Canadian circumpolar region and entire population, 2001 census (*1*)

Region	Total	Age, no. (%)				
		<2 y	2–4 y	5–19 y	20–64 y	>65 y
Circumpolar*	132,956	4,849 (3.7)	7,414 (5.6)	37,431 (28.2)	77,823 (58.5)	5,439 (4.1)
Canada†	30,007,095	652,120 (2.2)	1,044,160 (3.5)	6,082,585 (20.3)	18,339,680 (61.1)	3,888,550 (13.0)

*Yukon, Northwest Territories, Nunavut, northern Québec, and northern Labrador. †Includes the circumpolar region.

Canada has a universal healthcare system that includes access to both physician and hospital care. Publicly funded vaccination programs are a major component of disease control programs. Universal infant *H*. *influenzae* type b (Hib) vaccination programs were implemented in the Yukon, Northwest Territories, Nunavut, and northern regions of Québec and Labrador in the early 1990s. Pneumococcal polysaccharide vaccine, which protects against 23 serotypes of *S*. *pneumoniae*, has been available since 1983 and is recommended by the Canadian National Advisory Committee on Immunization (NACI) for all adults >65 years of age and children >2 years of age at high risk for infection. The 7-valent pneumococcal conjugate vaccine (PCV7) protects against 7 serotypes of *S*. *pneumoniae* and has been available in Canada since 2001; NACI recommends it for all children <2 years of age and children <5 years of age at high risk for disease. Meningococcal C conjugate vaccine has been recommended by NACI for all children <5 years of age, adolescents, and young adults since 2001 (*6*). Implementation of these NACI recommendations has occurred at various times throughout the region.

In Canada, communicable disease reporting is mandated at the provincial or territorial level; the list of reportable diseases varies by region. Reporting to national notifiable disease surveillance is not mandatory, and timely submission of case-by-case data with epidemiologic, clinical, and laboratory information is variable. Therefore, to increase the understanding of the epidemiology of invasive bacterial diseases in northern populations, Canada has participated in International Circumpolar Surveillance (ICS) since its inception in 1999. ICS is a population-based invasive bacterial disease surveillance network of circumpolar countries that includes the United States, Canada, Greenland, Iceland, Finland, Norway, and Sweden. We describe Canadian ICS data from 1999 through 2005, including the effect of universal PCV7 programs on invasive *S*. *pneumoniae* disease in children <2 years of age.

## Methods

### Case Reporting and Data Collection

Surveillance of invasive disease caused by *S*. *pneumoniae* began January 1, 1999. Surveillance for invasive *H*. *influenzae*, group A streptococci (GAS), group B streptococci (GBS), and *Neisseria meningitidis* commenced January 1, 2000. Cases reportable to ICS are defined as persons from whom an organism under surveillance is isolated from blood, cerebrospinal fluid, or other normally sterile site. Patients with clinical epiglottitis from whom *H*. *influenzae* is isolated from an epiglottis swab are also reportable to ICS. Cases are reported to public health officials by physicians or laboratories serving regions under surveillance; this includes patients managed outside of the region. Unconfirmed cases are not included. Standardized case report forms are completed in the region by trained communicable disease officers and include demographic, clinical, vaccination, and risk factor information. For the vaccine-preventable diseases (caused by *S*. *pneumoniae*, Hib, and *N*. *meningitidis*), details on the type of vaccine received are not currently available; however, information on the number of doses received is available. Reference laboratory representatives and communicable disease officers from each region participate in quarterly and annual data audits to ensure completeness of case finding and reporting.

### Laboratory Methods

A network of laboratories ascertains infection with any of the 5 organisms under surveillance within the region. Invasive isolates are submitted to 1 of 3 Canadian reference laboratories (National Centre for Streptococcus, National Microbiology Laboratory, and Laboratoire de Santé Publique du Québec). The reference laboratory confirms the isolate’s identity, determines its serotype or serogroup, and tests for antimicrobial susceptibility. Laboratories also participate in an ongoing quality control program.

Isolates were confirmed as *S*. *pneumoniae* by using conventional methods of identification (*7*). Strains were classified by the capsular swelling technique (*8*,*9*) by using commercial antisera (Statens Serum Institut, Copenhagen, Denmark). Antimicrobial drug susceptibility testing was performed by using the broth microdilution method consistent with National Committee for Clinical Laboratory Standards guidelines current at the time of testing (*10*,*11*).

M typing was performed on all submitted GAS isolates according to standardized methods (*12*) by using M type–specific antisera prepared in-house. Antisera to 61 of 86 internationally recognized M types, representing the most common M types (*13*), were available; strains for which an M type could not be assigned were classified as M nontypeable. GBS serotyping was performed according to conventional serologic techniques (*14*) by using type-specific antisera prepared in-house. Antisera were available for all 9 internationally recognized serotypes (Ia, Ib, II, III, IV, V, VI, VII, and VIII). For both organisms, Lancefield hot-acid extracts were prepared from the clinical isolates and tested in Ouchterlony immunodiffusion agar slides with appropriate control strains.

*H*. *influenzae* was confirmed by standard biochemical tests (*15*), and biotypes were determined according to current nomenclature (*16*). Serotyping was conducted by using a slide agglutination assay with antisera from commercial sources (Difco, Oakville, Ontario, Canada, and Denka Seiken, Tokyo, Japan). *N*. *meningitidis* was identified by using standard biochemical tests (*17*). Serogrouping was conducted by using bacterial agglutination with rabbit antisera to the different serogroups. Serotyping and serosubtyping were conducted by using an indirect, whole-cell, ELISA with monoclonal antibodies (*18*).

### Statistical Analysis

Statistical analysis was conducted by using SAS statistical package version 9.1 (SAS, Cary, NC, USA). The analyses were stratified by organism. Crude and age-specific annual incidence was calculated by using total and age-specific population estimates from the Demography Division of Statistics Canada for 2001 (*1*). Because of the small numbers of cases per year, 3-year period–based annual incidence rates were calculated for each organism to determine time trends. Rates were calculated for 2 periods (1999 for *S*. *pneumoniae* or 2000 for *H*. *influenzae*, GAS, and GBS to 2002 and 2003–2005). Regression analysis was conducted to detect trends in crude annual incidence rates over time. Crude incidence rates by ethnicity were calculated by using population data from the aboriginal population profile, which was developed from 2001 census data (*2*). Because of the lack of additional period estimates, no trend analyses were conducted on data by ethnicity.

Information about PCV7 program implementation was collected from the regions (Table 2). The prevaccination period was defined as 1999–2002, and the program implementation period was defined as 2003–2005. Because Labrador implemented its vaccination program during the second period, data from this region on the effect of the vaccine were excluded from the analysis. Sensitivity analyses that included data from Labrador in both arms was conducted to ensure this did not alter the results. Annual incidence of *S*. *pneumoniae* for all ages and the number of cases in children <2 years of age were compared for the 2 periods. Bivariate analysis was conducted by using χ^2^ and Fisher exact tests.

**Table 2:** Implementation dates of infant vaccination programs with universal 7-valent pneumococcal conjugate vaccine, by circumpolar region, Canada

Region	Date
Northern Québec	Apr 2002
Nunavit	Sep 2002
Northern Labrador	Jul 2003
Yukon	Jun 2005
Northwest Territories	Jan 2006

## Results

There were 251 confirmed cases of invasive disease caused by *S*. *pneumoniae* in northern Canada from 1999 through 2005. During 2000–2005, 62 cases of invasive disease caused by *H*. *influenzae*, 45 caused by GAS, 17 caused by GBS, and 6 caused by *N*. *meningitidis* were reported. Because of the small number of *N*. *meningitidis* cases reported, no further disease-specific analyses were conducted for this organism.

### Cases and Incidence Rates

In the ICS region, the crude annual incidence rate for *S*. *pneumoniae* was highest in 2001 (38.4/100,000 population) and lowest in 2005 (17.3/100,000 population), but this downward trend was not statistically significant (p = 0.119 by F test for slope). The age-specific incidence rate in children < 2 years of age decreased during 2000–2004 but increased in 2005. However, these incidence rates are based on a small number of cases and changes in rates over time should be interpreted with caution. The incidence rates in the population >65 years of age, who were eligible for the 23-valent polysaccharide vaccination, did not show any trend (Table 3). During 1999–2002 and 2003–2005, the crude annual incidence rates were 34.0 and 23.6/100,000 population/year, respectively. Although this finding suggests a decreasing incidence over the 2 periods, data from additional periods are necessary to determine if this is reflective of a trend.

**Table 3:** Crude annual incidence of *Streptococcus pneumoniae* in the Canadian circumpolar region by age group, 1999–2005

Year	Incidence/100,000 population (no. cases)		
	Total population	Age <2	Age >65
1999	25.6 (34)	226.9 (11)	55.2 (3)
2000	33.8 (45)	226.9 (11)	36.8 (2)
2001	38.4 (51)	165.0 (8)	91.9 (5)
2002	26.3 (35)	123.7 (6)	73.5 (4)
2003	22.6 (30)	103.1 (5)	110.3 (6)
2004	24.8 (33)	61.9 (3)	36.8 (2)
2005	17.3 (23)	165.0 (8)	73.5 (4)

Among the 240 (95.6%) of 251 *S*. *pneumoniae* cases with serotype information, the most common serotypes were type 1 (30.4%), type 8 (8.8%), type 14 (7.9%), type 4 (6.3%), and type 6B (5.8%). A total of 47 (60%) of 52 cases in children <2 years of age were caused by PCV7 serotypes. Among persons >65 years of age, 23 (88.5%) of 26 cases were caused by serotypes in the polysaccharide pneumococcal vaccine.

There were no trends in overall crude annual incidence rates of *H*. *influenzae* or GBS (Table 4). The crude annual incidence rate of *H*. *influenzae* was lowest in 2003 (4.5/100,000 population) and highest in 2001 (13.5/100,000 population). Among 59 cases with serotype information, 31 (59%) were *H*. *influenzae* type a (Hia); 73.3% of these cases were in children <2 years of age. Eight cases (13.6%) of Hib were reported during the surveillance period: 6 in infants <5 months of age, 1 in a child 18 months of age, and 1 in an adult. Two infants had no vaccine information. The adult and 1 infant had not been vaccinated; the remaining 4 children had received only 1 Hib dose. Thus, none of these cases were considered vaccine failures. GAS incidence increased significantly during 2001–2005 (*F* = 229.371, p = 0.01). The largest number of cases (n = 14) was reported in 2005 with a crude incidence rate of 10.5/100,000 population. The increase in GAS cases was not clustered by region, period, or serotype. A total of 1–4 cases of GBS were reported in the region annually, for a crude annual incidence range of 0.8–3.0/100,000 population.

**Table 4:** Crude annual incidence of *Haemophilus influenzae*, GAS, and GBS in the Canadian circumpolar region, 2000–2005*

Year	Incidence/100,000 population (no. cases)		
	*H. influenzae*	GAS	GBS
2000	6.0 (8)	5.3 (7)	3.0 (4)
2001	13.5 (18)	1.5 (2)	3.0 (4)
2002	6.0 (8)	3.8 (5)	1.5 (2)
2003	4.5 (6)	5.3 (7)	3.0 (4)
2004	8.3 (11)	7.5 (10)	1.5 (2)
2005	8.3 (11)	10.5 (14)	0.8 (1)

*GAS, group A streptococci; GBS, group B streptococci.

### Demographic Characteristics

Infections with *S*. *pneumoniae*, *H*. *influenzae*, and GAS were more common in male patients (59.8%, 58.3%, and 62.2%, respectively). Seventy-one percent of all cases of GBS were among female patients; 17.6% (3/17) of cases of GBS were among newborns <1 month of age. All of the newborn cases occurred in the early neonatal period. These 4 organisms disproportionately affect children <2 years of age and persons >65 years of age. Although <4% of the surveillance population was <2 years of age, 21%–67% of the infections occurred within this age group. Similarly, adults >65 years of age had a higher proportion of cases than the surveillance population they represent (Figure).

**Figure:**
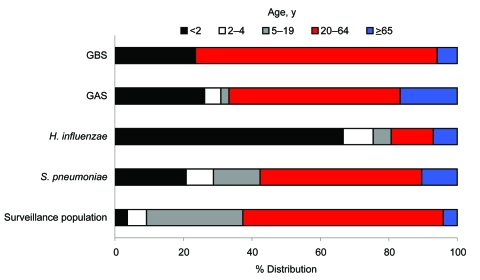
Age distribution of surveillance population and cases of infection with group B streptococci (GBS), group A streptococci (GAS), *Haemophilus influenzae*, and *Streptococcus pneumoniae* in the Canadian circumpolar region.

Data on patient ethnicity was missing for 42 (11.0%) of 381 cases of invasive bacterial disease. Aboriginal persons represented 59% of the surveillance population and 84% of cases of *S*. *pneumoniae*, 92% of *H*. *influenzae*, 93% of GAS, and 53% of GBS. To assess changing incidence over time by ethnicity, the surveillance period was divided into 2 periods (1999–2002 and 2003–2005 for *S*. *pneumoniae* and 2000–2002 and 2003–2005 for all other organisms). For all but GBS, the crude annualized incidence rates were higher in the aboriginal population than in the non-aboriginal population (Table 5). For *S*. *pneumoniae*, the disparity between aboriginal persons and non-aboriginal persons decreased from 4.6-fold to 2.5-fold between the 2 periods. Among aboriginal persons, the GAS rate in the second period was nearly double that seen in the first period. Six of 8 case-patients with Hib and all 27 case-patients with Hia for whom ethnicity data were available were among aboriginal persons.

**Table 5:** Crude annualized incidence of *Streptococcus pneumoniae, Haemophilus influenzae,* GAS, and GBS by ethnicity in the Canadian circumpolar region, 1999–2005*

Organism, ethnicity	Incidence/100,000 population/year (no. cases)	
	1999–2002	2003–2005
*S. pneumoniae*		
Non-aboriginal	9.6 (20)	10.2 (16)
Aboriginal	44.2 (134)	25.1 (57)
	2000–2002	2003–2005
*H. influenzae*		
Non-aboriginal	0.6 (1)	1.9 (3)
Aboriginal	11.9 (27)	8.4 (19)
GAS		
Non-aboriginal	0	1.9 (3)
Aboriginal	5.7 (13)	11.0 (25)
GBS		
Non-aboriginal	2.6 (4)	1.9 (3)
Aboriginal	2.2 (5)	1.3 (3)

*GAS, group A streptococci; GBS, group B streptococci.

### Clinical Findings and Outcomes

Information on clinical findings was available for 380 of 381 cases (Table 6). The most common primary clinical finding for invasive *S*. *pneumoniae* was pneumonia (64.5%), followed by bacteremia/septicemia (21.5%). Among invasive GAS cases, the most common primary clinical finding was cellulitis (31.1%); necrotizing fasciitis accounted for 11.1%. For cases with *H*. *influenzae* or GBS, the most common primary clinical findings were bacteremia/septicemia (33.9% and 47.1%, respectively). There were no reported cases of epiglottitis caused by *H*. *influenzae*.

**Table 6:** Primary clinical symptoms for patients infected with *Streptococcus pneumoniae, Haemophilus influenzae*, GAS, or GBS in the Canadian circumpolar region*

Symptoms	*S. pneumoniae* (n = 251), no. (%)	*H. influenzae* (n = 62), no. (%)	GAS (n = 44),† no. (%)	GBS (n = 17), no. (%)
Septicemia/bacteremia	21.5 (54)	33.9 (21)	22.7 (10)	47.1 (8)
Pneumonia	64.5 (162)	21.0 (13)	13.6 (6)	0
Meningitis	6.0 (15)	27.4 (17)	0	11.8 (2)
Cellulitis	0.8 (2)	4.8 (3)	31.8 (14)	11.8 (2)
Empyema	3.2 (8)	3.23 (2)	2.3 (1)	0
Necrotizing fasciitis	0	0	11.4 (5)	0
Septic arthritis	0.8 (2)	4.8 (3)	9.1 (4)	17.7 (3)
Other‡	3.2 (8)	4.8 (3)	9.1 (4)	11.8 (2)

*GAS, group A streptococci; GBS, group B streptococci. †One patient had no information recorded for clinical symptoms. ‡Includes peritonitis, appendicitis, pericarditis, osteomyelitis, and endocarditis.

Cases with GAS had the highest case-fatality rate (18.2%, 8 of 44 cases with outcome data). Two (40%) of 5 cases with GAS and necrotizing fasciitis resulted in death; however, this difference was not statistically significant (p = 0.065, by Fisher exact test). The case-fatality rates for infections with the other 3 organisms were 4.8% (11/230) for *S*. *pneumoniae*, 6.1% (3/49) for *H*. *influenzae*, and 7.1% (1/14) for GBS. The relative risk for death did not vary by ethnicity (p = 0.550, by Fisher exact test).

### Effect of PCV7 Immunization Programs

Fifty-two cases of S. *pneumoniae* occurred in children <2 years of age. Eight of these case-patients had received >1 dose of pneumococcal vaccination: 1 case-patient with of PCV7-preventable *S*. *pneumoniae* had received only 1 dose of vaccine (serotype 6b), 6 case-patients had serotypes that were not preventable with PCV7 (serotypes 19A, 20, 13, 15a, 22, and 22F), and 1 case-patient had no information on serotype. These findings suggest that there were no known cases of vaccine failure.

Numbers of cases of disease caused by *S*. *pneumoniae* in children <2 years of age were compared during the prevaccination and program implementation periods (Table 7). In regions where universal PCV7 infant programs were implemented in 2002 (Nunavut and northern Québec), 19 cases with PCV7 serotypes were reported during the prevaccination period and no cases were reported during the program implementation period. In the other Canadian ICS regions where universal PCV7 infant programs were implemented after 2002 (excluding Labrador), 6 cases of PCV7-preventable *S*. *pneumoniae* disease occurred in the prevaccination period and 3 cases in the program implementation period. A χ^2^ analysis showed that the number of cases of PCV7-preventable illness by vaccination region was statistically significant (p = 0.019, by Fisher exact test). These results are conservatively biased because PCV7 was available in all regions in the program implementation period and a universal vaccination program was started during the later half of 2005 in the Yukon, which may have reduced the number of cases seen in the comparison area. Our findings suggest that early implementation of universal PCV7 programs was associated with a reduction in PCV7-preventable illness in children <2 years of age. A sensitivity analysis including Labrador in both arms (program or no program) did not change the statistical significance of the difference because 1 case in Labrador was not vaccine preventable.

**Table 7:** Effect of universal PCV7 programs for children <2 y of age in the Canadian circumpolar region*

Location, period	No. cases with PCV7 serotypes	No. cases without PCV7 serotypes	Total
Northern Québec and Nunavut			
Prevaccination (1999–2002)	19	5	24
Program implementation (2003–2005)	0	8	8
Total cases	19	13	32
Northwest Territories and Yukon			
Prevaccination (1999–2002)	6	2	8
Program implementation (2003–2005)	3	3	6
Total cases	9	5	14

*PCV7, 7-valent pneumococcal conjugate vaccine.

## Discussion

To our knowledge, this study is the first comprehensive surveillance report on invasive bacterial diseases in the Canadian Arctic. Disease caused by *S*. *pneumoniae* continues to be a serious problem in northern Canada. The annual rate for 2003–2005 was 23.6/100,000 population/year, which is more than twice the reported rate of invasive pneumococcal disease in the overall Canadian population (9.1/100,000 population in 2004) (*19*). Although this rate is lower than that seen in the earlier period (1999–2002, 34.0/100,000 population), additional data will be needed to determine if the decreasing trend is sustained. The decrease in the disease incidence may be partly attributed to PCV7 programs, as well as the mass pneumococcal polysaccharide vaccination campaigns launched in 2001 and 2002 in response to outbreaks of serotype 1 disease in parts of the region, which reduced the occurrence of this predominant serotype in subsequent years (*20*,*21*). Reduction in the number of cases among children <2 years of age in regions where universal infant PCV7 programs were implemented in 2002 (northern Québec and Nunavut) is an early indicator of the effect of the vaccination program. This finding is likely a conservative assessment of the effect of the program, given the staggered implementation of universal vaccine programs in the circumpolar region.

Although aboriginal persons represented 84% of invasive *S*. *pneumoniae* cases, a substantial reduction in disease incidence was demonstrated in the program implementation period. Progress toward elimination of this health disparity has also been reported for indigenous populations in Alaska (*22*) and Australia (*23*), where PCV7 has been available since 2001 to all indigenous children <2 years of age. The incidence of *S*. *pneumoniae* may be expected to decrease further among young children throughout northern Canada, particularly in aboriginal children, as universal PCV7 programs become fully implemented with sustained high coverage rates.

In contrast, there continues to be a health disparity for invasive *H*. *influenzae* disease. Annual period incidence rates for *H*. *influenzae* during 2003–2005 were >4-fold higher among aboriginal persons than among non-aboriginal persons. Although Hib disease is rare because of universal Hib vaccination, the greatest number of cases occurred among aboriginal persons, a group known to be at increased risk for Hib disease (*24*–*26*). Studies in Alaskan aboriginal populations suggest that continued low-level nasopharangeal colonization facilitates transmission to susceptible children (*4*). Environmental and housing conditions, including overcrowding, are also potential contributing factors to these health disparities (*27*–*29*). The data also indicate an apparent emergence of type a disease, with all Hia cases occurring among aboriginal persons. Hia disease has also been reported in aboriginal populations in the United States and Australia (*30*–*32*). A possible shift in disease epidemiology to non-b serotypes has been suggested from findings in an adjacent Canadian region (*33*), whereas a sustained increase in non-b serotypes has not been detected in Alaska (*4*).

The incidence of GAS in the ICS region has been increasing since 2001. Although changes in rates over time should be interpreted with caution because the number of cases is small, this apparent increase in GAS disease is being monitored. The rate in northern regions is greater than that in the overall Canadian population. In 2004, the incidence of GAS in the ICS population was 7.5 compared with the Canadian rate of 2.7/100,000 population (Public Health Agency of Canada, unpub. data). Aboriginal persons represented the greatest proportion of GAS cases; this has also been observed among indigenous populations in the United States (*34*) and Australia (*35*). As with *S*. *pneumoniae* and *H*. *influenzae*, this may be partially attributed to poverty and crowded living conditions in these populations (*36*).

A major limitation of the data is that the number of reported cases is too small to permit analysis of smaller areas and subpopulations within the region. In addition, changes in rates over time should be interpreted with caution due to small numbers of cases. It is also expected that the number of reported cases of invasive bacterial diseases is an underestimate. However, the enhanced nature of the surveillance system and regular data audits represent improvements over routine passive surveillance. Laboratory specimens may not have been taken before initiation of empiric treatment, and collection and transportation of clinical specimens are difficult in remote areas experiencing extreme temperatures (*20*,*21*). Unfortunately, because vaccine registries have not yet been fully implemented in Canada, immunization coverage rates are not available. This situation limits our ability to evaluate the effect of vaccination programs.

Despite these limitations, data collected by ICS contribute to understanding the epidemiology of invasive bacterial diseases among northern populations in Canada and throughout the world. These data assist in formulation of prevention and control strategies, including immunization recommendations (*6*). ICS data have also been instrumental in identifying potentially emerging pathogens such as Hia. Continued collection of data will be used to assess the effect of vaccination in this population and monitor serotype replacement, antimicrobial drug resistance, and reductions in disparities in Northern populations.
